# Advancing infection prevention and control through artificial intelligence: a scoping review of applications, barriers, and a decision-support checklist

**DOI:** 10.1017/ash.2025.10191

**Published:** 2025-11-25

**Authors:** Silvana Gastaldi, Ermira Tartari, Giovanni Satta, Benedetta Allegranzi

**Affiliations:** 1 Department of Infectious Diseases, Epidemiology, Biostatistics and Mathematical Modeling Unit (EPI) Istituto Superiore di Sanità, Italy; 2 Faculty of Health Sciences, University of Malta, Msida, Malta; 3 Centre for Clinical Microbiology, University College London, London, UK; 4 Department of Communicable Diseases, Global Lead for Infection Prevention and Control, World Health Organization, Eastern Mediterranean Regional Office, Cairo, Egypt

## Abstract

**Objective:**

To examine how artificial intelligence (AI) has been applied to infection prevention and control in healthcare, identify barriers and risks affecting implementation, and develop a structured checklist to support safe adoption.

**Design:**

Scoping review conducted in line with Joanna Briggs Institute methodology and reported according to PRISMA-ScR.

**Methods:**

PubMed, Scopus, and Web of Science were searched for primary studies (2014–2024) describing real-world AI applications for IPC. Studies reporting implementation experiences, outcomes, or risks were included. Data on study design, AI type, IPC function, integration level, barriers, and outcomes were extracted and synthesized thematically to derive a 41-item decision-support checklist.

**Results:**

Of 2,143 records screened, 100 studies met inclusion. Most were published since 2022, with the United States and China leading output. Machine learning dominated (75%), mainly for predictive analytics (53%), HAI detection (13%), and hand hygiene monitoring (13%). Only 15% of tools were integrated into existing digital infrastructures. Barriers centred on data quality (45%), technical and data related (16%), and economic/technical constraints (16%). Reported risks clustered around operational failures (35%), technical errors (33%), and data security (12%). Evidence was heavily skewed toward high-income countries, with limited prospective validation or implementation science.

**Conclusions:**

AI offers clear promise for IPC, particularly in early detection and compliance monitoring, but its translation into practice remains constrained by data fragmentation, limited integration, and uneven readiness across settings. Our evidence-informed checklist provides IPC teams with a structured tool to assess feasibility, governance, and resource needs before adoption, supporting safer and sustainable innovation.

## Introduction

Healthcare-associated infections (HAIs) remain a major threat to patient safety and quality of care worldwide (WHO, 2022).^1^ Conventional IPC measures, such as active surveillance, outbreak investigations, and transmission-based precautions, are essential but often reactive and resource-intensive.^1^

Artificial intelligence (AI) has emerged as a promising tool to enhance IPC by linking diverse data sources, including laboratory results, resistance profiles, patient movement, and compliance behaviors.^2,3,4^ Applications include predictive analytics, infection detection, hand hygiene monitoring, and compliance auditing. While evidence demonstrates technical feasibility, translation into everyday practice remains limited due to fragmented data, poor integration, and organizational or ethical concerns.^5,6^

This review synthesizes evidence on real-world AI applications for IPC, identifies barriers and risks, and develops an evidence-informed readiness checklist to support an easier and safer adoption. The checklist aligns empirical findings with international governance frameworks, such as the WHO ethics guidance on AI in health^6^ and the EU Artificial Intelligence Act.^7^

## Methods

This review followed the Joanna Briggs Institute (JBI) methodology for scoping reviews^8^ and is reported in line with PRISMA-ScR guidelines.^9^ We addressed two questions: (1) What are the documented applications, barriers, and real-world outcomes of AI in IPC? and (2) How can these findings inform a structured checklist for feasibility, risk, and organizational readiness?

Eligible studies were primary research (prospective cohorts, retrospective analyses, pilots, multicenter trials) describing AI applications in healthcare IPC with practical outcomes (eg, effectiveness, feasibility, workflow integration, risks). We excluded theoretical models, algorithm-only studies without real-world application, and non-peer-reviewed material such as editorials or conference abstracts. Digital health tools or Internet of Things (IoT) solutions were included only if they incorporated an AI component relevant to IPC functions. Searches covered PubMed, Scopus, and Web of Science (period Jan 2014–Dec 2024; last search Feb 2025) using a Population–Concept–Context framework.^8^ No language restrictions were applied. Search terms are detailed in Supplementary Appendix A.

**Figure 1.**
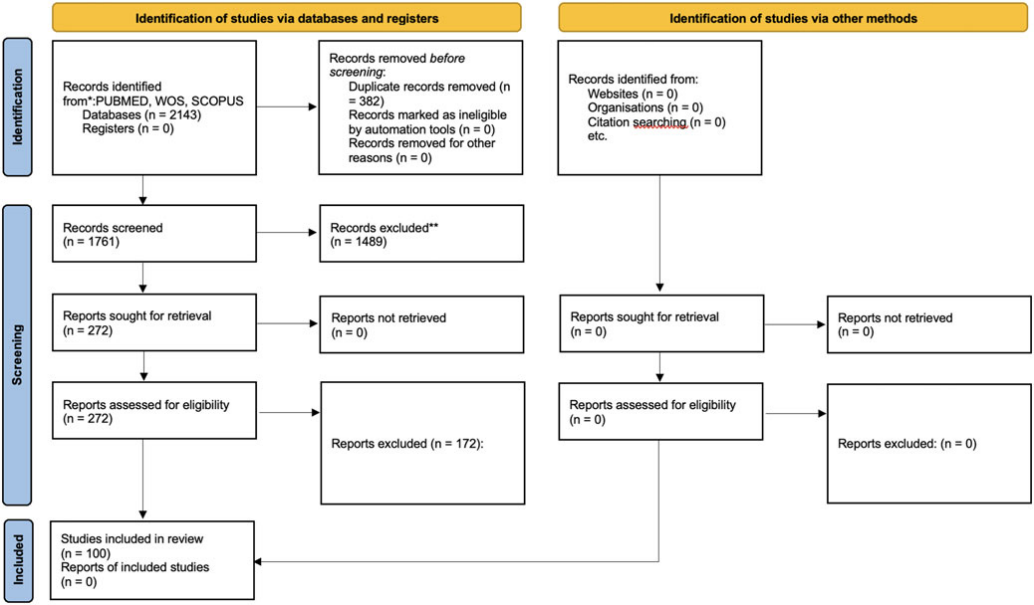
PRISMA 2020 flow diagram for the scoping review.

Records were screened in Rayyan^10^ tool by two reviewers (SG, ET) with adjudication by a third (GS). The PRISMA flow diagram (Figure 1) summarizes the selection. Data were extracted into a piloted template and charted in Excel. Variables included study characteristics, AI type, IPC application, implementation features, outcomes, barriers, and risks. Two reviewers (SG, ET) cross-validated entries.

Data synthesis combined descriptive and thematic analysis. Descriptive synthesis grouped studies by AI type and IPC function; thematic synthesis identified implementation barriers, risks, and facilitators. Rooted on this analysis, we developed a 41-item readiness checklist, mapped to six domains: governance and policy, data quality and interoperability, technical and infrastructure, human and workflow, economic and resource, and risk and compliance. The full data set, including structured classifications by AI method, IPC focus, implementation features, and risks, is available in Supplementary Appendix B. Each study in the following tables retains the same numeric identifier as in Appendix B, allowing readers to cross-reference study characteristics with the full citation.

## Results

Out of 2,143 identified records, 382 duplicates were removed. Of the remaining 1,761 titles and abstracts screened, 272 full-text articles were reviewed. A total of 100 studies met all inclusion criteria and were included in the final study.

Of the 100 studies included in this review, only one (1%) was published before 2017. In contrast, 31% were published between 2017 and 2021, while more than two-thirds (68%) appeared between 2022 and 2024, indicating an acceleration in research activity in recent years.

Geographically, the evidence was mostly generated in high-income settings. The United States accounts for 35% of studies, primarily focused on predictive analytics. China contributed 17%, often exploring deep-learning approaches. European countries represented the 19%, with emphasis on workflow integration and hospital-acquired infection (HAI) surveillance. The Asia-Pacific region outside China added another 14% and Latin America contributed 5%. A small number of studies (6%) originated from sub-Saharan Africa and the Middle East, reflecting emerging research capacity but limited scale.

Using the four-tier scheme (Experimental, Interventional/Implementation, Retrospective, and Validation) applied to the 100 eligible papers in Table 1, almost half of the evidence base remains anchored in retrospective work (49%), which relied on existing health or laboratory data to build or test models.

**Table 1.** Design standardized classification distribution

Design standardized classification	Description	%	References
Experimental	Algorithm development or simulation, model development, Building or stress-testing an algorithm, usually offline on historic data.	30%	^15,17,18–21,27, 29–32,34,36,40, 41,44,46,47,51,60,62,65, 66,67,69,96,100,104,106,110^
Interventional/implementation	Real-world pilots, deployment, or prospective testing, cross-sectional.	17%	^11,14,49,55,56,63,68,80, 90,92,94,102,103,105,107,108, 109^
Retrospective	Non-interventional studies using historic patient data, Performance assessment against gold standards.	49%	^12,13,16,22, 23–26,28,33,35, 37,38,39,42,43,45,48,50, 52–54,57–59,61,64, 70–73,75,78,79, 81–85,87,88,89, 91,93,95,97–99, 101^
Validation studies	Model development with internal validation and external validation.	4%	^74,76,77,86^

Experimental designs accounted for 30%, focusing on technical feasibility in controlled settings. Far fewer studies examined real-world use: 17% reported interventional or implementation pilots, while only 4% carried out independent validation before wider deployment.

### Types of AI technology

A full breakdown of AI techniques and corresponding references is available in Table 2 and detailed in Supplementary Appendix B.

**Table 2.** AI technology categories distribution

AI technology	(%)	References
Machine learning	75	^12–16,19,20,22–30, 33,34,35,37–40, 42–48,50,54,55, 60,61,65,67, 68,70,71,73–92, 94–103,106,108,110^
Deep learning	21	^17,18,21,32, 36,44,51–53,62–64, 66,69,72,93,96,104,105,107, 109^
Generative AI	4	^11,31,41,49^

Machine learning (ML) was the dominant technique, used in 75% of the 100 studies. This technique often relies on supervised algorithms such as logistic regression, random forests, or gradient boosting to support tasks like infection risk prediction and HAIs surveillance based on EHRs or sensor data. For more details, see next section on IPC applications blocks.

Deep learning approaches appeared in 21 studies (21%) and included for example the use of convolutional neural networks (CNNs) technologies for surface contamination detection^60^ but also on video-based hand hygiene monitoring,^63^ surgical site infections (SSI),^96^ and early detection of multidrug-resistant infections.^52^

Generative AI was used in just four studies (4%). These projects piloted large language models (LLMs) for IPC education,^11^ policy summarisation^31^ through expert consensus, HAI’s surveillance^41^ and Hand hygiene.^49^

### IPC applications supported by AI distribution

**Figure 2.**
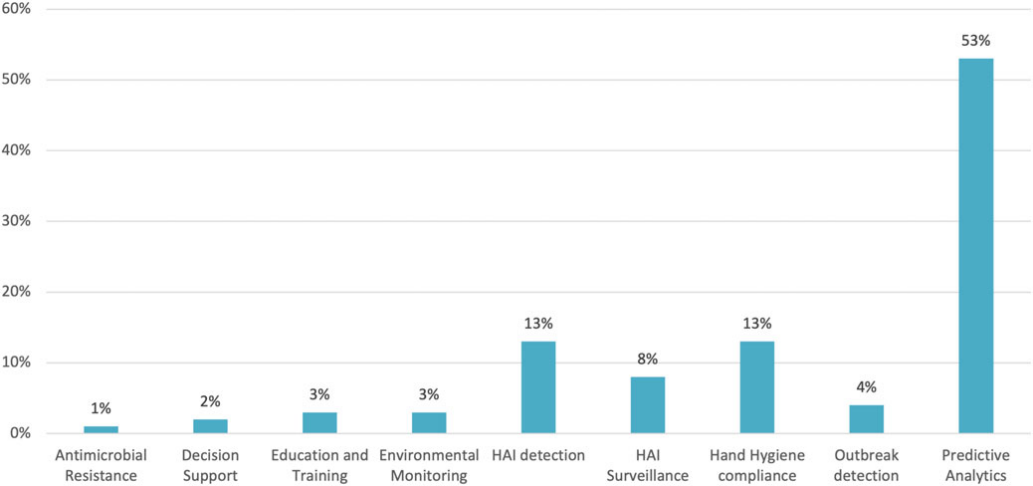
IPC applications distribution.

Among the 100 primary studies, nine distinct IPC application areas were identified (Table 3 and Figure 2).

**Table 3.** IPC applications supported by AI distribution

IPC application area	(%)	References
Antimicrobial resistance	1	^110^
Decision support	2	^31,59^
Education and training	3	^11,44,55^
Environmental monitoring	3	^26,69,106^
HAI detection	13	^18,19,36,60,64,76,77,79, 81,86,94,98,105^
HAI surveillance	8	^25,29,34,41,45,54,72,108^
Hand hygiene compliance	13	^17,21,32,37,40,47,49,51, 63,66,68,96,109^
Outbreak detection	4	^15,42,43,101^
Predictive analytics	53	^12–14,16,20,22–24, 26,28,30,33,35,38,39,46, 48,50,52,53, 56–58,61,62,65, 67,70,71,73–75, 78,80,82–85,87, 88–93,95,97,99, 100,102–104,107^

Predictive-analytics systems accounted for 53% of the studies. These papers typically trained supervised models such as logistic regression, gradient boosting, or recurrent networks on electronic-health-record data to forecast patient-level risks such as, for example, central-line associated bacteremia,^56,39^ Multi Drug Resistance (MDR)^35,52^ or SSI.^12,38,74,107^ In some cases, ward-level early-warning scores were generated by combining laboratory, vital-sign, and admission data streams.^56^

Hand hygiene compliance monitoring was reported in 13 studies (13%), with applications varying through video analytics using CNNs, badge-based proximity sensors,^17,96^ alcohol-dispenser sensors linked to compliance dashboards,^109^ and optical microscopy with ML classification for hand contamination.^40^

Thirteen studies (13%) focused on HAI detection, applying classification algorithms, microbiology, imaging, or pharmacy records to flag active infections such as SSI,^36,64,77,76,79^ central-line–associated bloodstream infection^94^ or urinary tract infections (UTIs).^86^ Many of these tools operated continuously in clinical workflows.^60,98^

Eight studies (8%) targeted HAI surveillance by aggregating time-series data for incidence-trend analysis.^25,54,108^ Automated case-finding rules were often calibrated against conventional manual surveillance^45^ but also using Natural Language Processing (NLP)^29,34,72,108^ and Generative AI.^41^ Outbreak-detection models were described in four studies (4%). Approaches ranged from supervised clustering of ward alerts^101^ to whole-genome-sequencing pipelines that feed resistance-related single-nucleotide-polymorphism data into transmission-mapping algorithms.^42,43^ One study integrated environmental IoT sensors to identify spatiotemporal hotspots.^15^

Less-common themes included education and training platforms (3 %), where generative- or retrieval-augmented language models generated scenario-based learning modules^11^ or PPE monitoring through computer vision founded on Human-AI Collaboration;^44,55^ environmental monitoring (3 %), employing image recognition or air-quality sensors for surface-cleanliness verification;^27,69,106^ and AMR prediction (1%), also using Plasmonic Nanosensors.^110^ Finally, decision-support dashboards (2 %) evaluating ChatGPT’s reliability in agreeing with expert statements^31^ or risk stratification for UTIs^59^ were described in two studies.

### Barrier profile

**Figure 3.**
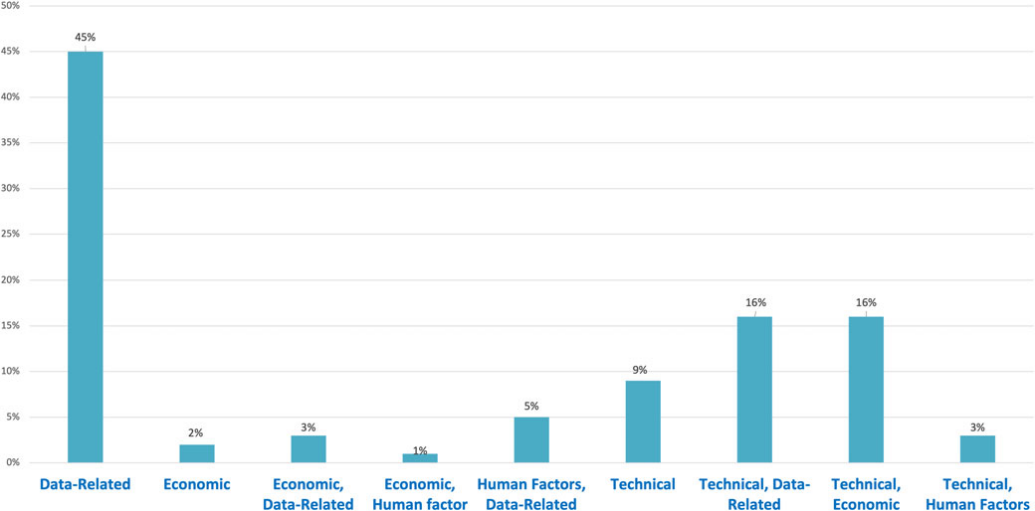
Barrier categories distribution.

A total of 4 barrier categories were coded across 100 primary studies, clustering into nine composite categories (Table 4 and Figure 3).

**Table 4.** Barrier categories

Barrier category	(%)	References
Data-related	45	^14,18,20,22–24, 26,28,31,41,48,53,56,57, 61–65,70–75,77,79, 80,84,86,87, 88–95,97–100,103,107, 108^
Technical, data-related	16	^11,16,17,19,29,30,33,50, 54,58,67,74,76,81,83,101^
Technical, economic	16	^32–35,37–40,42–45,55, 68,69,102,104,110^
Technical	9	^12,15,25,47,59,66,78,82, 106^
Human factors, data-related	5	^13,36,46,51,52^
Technical, human factors	3	^21,44,99^
Economic, data-related	3	^27,85,96^
Economic	2	^105,109^
Economic, human factors	1	^60^

Data-related barriers were the most common, reported in 45 studies (45%), with issues such as incomplete records, non-standard terminologies, and delayed feeds.^22,31,77,78^ Barriers combining technical and economic elements (16%) were also frequent. Examples included high setup and maintenance costs for AI surveillance tools,^35^ computational demands and complexity of real-time systems,^38^ and substantial infrastructure requirements for whole-genome sequencing pipelines.^42,43^ A further 16% of studies reported barriers blending integration challenges with data shortcomings. Purely technical problems, including outages, high computational requirements, and sensor failures, appeared in 9%. Human factors coupled with data gaps, such as low trust or alert fatigue, were noted in 5%. Less frequent were mixed technical-human (3%), economic-data (3%), stand-alone economic (2%), and economic-human (1%) barriers.

### Risk taxonomy

**Figure 4.**
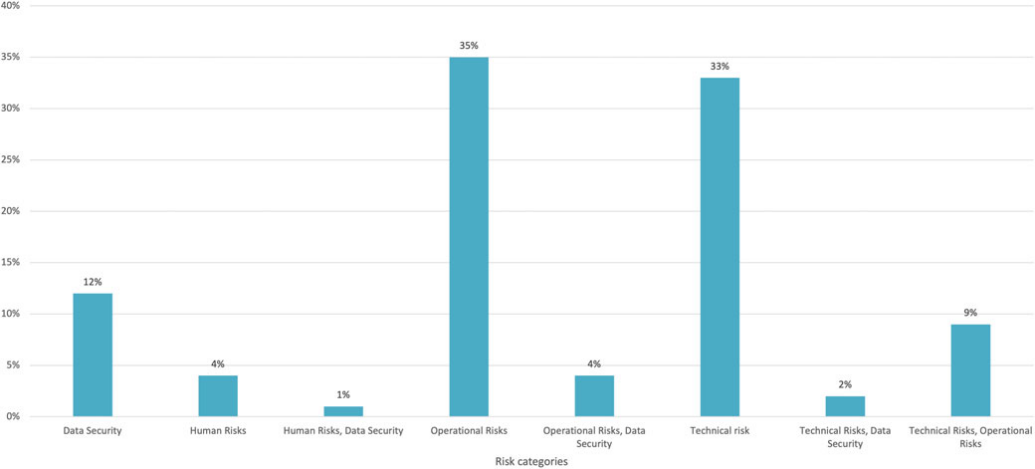
Risk categories distribution.

A total of 4 risk categories were coded across 100 primary studies, yielding eight composite categories (Table 5 and Figure 4). Risk reporting was highly granular, and many papers logged more than one category.

**Table 5.** Risk categories

Risk category	Percentage (%)	References
Operational risks	35	^11,12,16–18,23, 25,26,28,30,31,33,35,38, 42,44,48,54–59, 63,66,76,80–82, 84,85,90,97,99,107^
Technical risks	33	^13,14,20–22,24, 27,34,36,39,40,45,50,61, 62,64,65,67, 68,71–73,75,77, 78,83,86,88,89,91,92,101,108^
Data security	12	^15,37,60,69,70,74,79,96, 98,100,103,109^
Technical risks, operational risks	9	^41,43,53,87,93,94,104,105, 106^
Human risks	4	^46,95,102,110^
Operational risks, data security	4	^19,47,51,52^
Technical risks, data security	2	^29,32^
Human risks, data security	1	^49^

Operational issues were the most frequently documented risk type, recorded in 35 of 100 studies (35%). These papers described, for example, over-reliance on predictions without clinical confirmation^12,16^ or potential misinterpretation if model outputs are not validated.^18^

Technical failures followed closely, appearing in 33 studies (33%). Examples included server Risk of model overfitting^71,73^ or risk of under-detection in systems with incomplete digital documentation.^77,86^

Data-security concerns were reported in 12 studies (12%), typically focusing on patient or staff privacy regulations.^15,37,109^

Combined technical-and-operational risks featured in nine studies (9 %), while paired operational-and-data-security risks were noted in four studies (4 %).

Pure human-factor risks, such as over-reliance on AI outputs^110^ or diminished vigilance during downtimes,^102^ were documented in four studies (4 %). Two papers (2 %) combined technical and data-security risks,^29,32^ and one study (1 %) reported a mixed human-and-data-security category. ^49^

Digital-integration status

Of the 100 studies reviewed, only 15 papers (15 %) described AI tools already integrated into another digital layer of hospital or public health information systems. The main integration pathways were:

- **Automated surveillance shells**, documented in 4 studies (4 %), which connected AI analytics to existing IPC dashboards.^77,78,86,98^
- **IoT data streams**, featured in 2 studies (2 %), linking badge or environmental-sensor feeds to analytic engines.^15,60^
- **Whole-genome sequencing pipelines**, reported in 2 studies (2 %), integrating AI outbreak detection into genomic workflows.^42,43^
- **EHR-embedded models**, described in 2 studies (2 %), integrating real-time AI tools directly into electronic health record systems to enable dynamic risk prediction and decision support within clinical workflows.^58,79^

Furthermore, single studies (each 1 %) detailed integration via mHealth apps,^13^ computer-vision systems,^44^ computational-fluid-dynamics models,^106^ wearable devices^109^ and plasmonic nanosensors.^110^

The remaining 85 studies (85 %) reported prototypes without wider digital connectivity.

### Checklist refinement and practical use

To help IPC teams assess readiness for adopting AI tools, we translated the findings of this review into a structured, evidence-informed checklist (Table 6). The checklist contains 41 items grouped into six domains reflecting the most common barrier clusters identified across the literature: Governance and Policy, Data Quality and Interoperability, Technical and Infrastructure, Human and Workflow, Risk and Compliance and Economic and Resource.

**Table 6.** Structured readiness checklist for AI implementation in IPC

Domain	Item	Priority	Maturity (0–3)
Governance and policy	Is a multidisciplinary AI/IPC steering committee for oversight and strategic governance in place?	Medium	
	Has the AI tool been assessed against relevant AI-as-a-medical-device regulations and obtained the necessary clearance or documented exemption?	High	
	Is the AI tool aligned with the organization’s IPC goals or performance indicators?	High	
	Has patient/public representation been included in oversight of AI use in IPC?	Medium	
Data quality and interoperability	Are large, IPC-relevant, high-quality labeled datasets available for at least 12 months?	High	
	Are key data elements (e.g., admission date, microbiology results, device exposure) ≥95 % complete for the last 12 months?	High	
	Do source systems share a standard vocabulary (e.g. International Classification of Diseases ICD-10) or have a mapped crosswalk?	High	
	Is there a person in charge (data-governance steward) of making sure the data is accurate and managed properly?	Medium	
	Is data refreshed at least hourly (or near-real-time if required)?	High	
Data quality and interoperability	Does the institution provide HL7-FHIR (Health Level Seven - Fast Healthcare Interoperability Resources) or similar APIs (Application Programming Interfaces) for two-way data exchange?	High	
	Are compute, network and storage resources available and budgeted for peak loads?	High	
	Is a rollback plan in place for model/interface updates?	Medium	
	Can anonymized data or AI outputs be shared securely with external IPC/regional surveillance networks?	Medium	
Technical and Infrastructure (implementation and organizational readiness)	Has the model reached acceptable discrimination (e.g. AUC>.80) in external validation?	High	
	Does the AI tool provide explanations (like SHapley Additive exPlanations - SHAP values or saliency maps) that help clinicians understand its decisions?	Medium	
	Vendor maturity—Is the supplier able to provide implementation support and updates for>5 years?	Medium	
	Is a multidisciplinary implementation team for operational delivery (IPC, IT, data science, clinicians) in place?	High	
	Is end-user training and downtime protocol defined?	Medium	
Technical and infrastructure	Is roll-out staged (pilot - limited - full scale)?	Medium	
	Are roles/responsibilities for errors clearly assigned (vendor vs hospital)?	High	
	Are training, SOPs and communication plans defined?	Medium	
Human and workflow fit	Are adequate resources, staff expertise, and leadership support in place to adopt the AI solution?	High	
	Does the tool explain each alert or prediction in plain language?	Medium	
	Has expected alert volume been stress-tested?	Medium	
	Will outputs display inside dashboards staff already use?	High	
	Are standard operating procedures (SOPs), training materials, and staff communication plans defined and validated?	Medium	
	Is a change-management strategy in place (phased roll-out, communication, adoption KPIs)?	High	
Risk and compliance	Has the AI tool been classified under EU AI Act risk levels or FDA medical device categories?	High	
	Does the deployment comply with local data-protection laws and encrypt data in transit and at rest?	High	
	Is a contingency plan ready for system outages (manual override)?	High	
	Is continuous performance monitoring with drift alerts in place?	High	
	Is there evidence of harm mitigation strategies (alerts, human-in-the-loop)?	High	
	Does the system implement de-identification (removal of personal identifiers), encryption (data protection through secure coding), and IEC 81 001-5-1 cyber-controls (International Electrotechnical Commission standard for cybersecurity in health software)?	High	
	Is a documented model-monitoring and re-validation protocol in place (e.g., drift-detection thresholds, scheduled performance audits, retraining triggers, rollback procedures)?	High	
Risk and compliance	Have dedicated cybersecurity safeguards been implemented and verified for the AI system (network segmentation, encryption in transit/at rest, penetration testing, Operating System OS-patch policy)?	High	
	Were datasets checked for representativeness (e.g. minority groups)?	High	
	Are model design, training data, and decision logic documented in a way that allows independent audit?	High	
Economic and resource	Are funding streams secured to support maintenance, updates, and scale-up to new IPC use-cases over time?	High	
	Are IT, compute, and staffing needs adequately resourced within the allocated budget?	High	
	Has a total-cost-of-ownership (TCO) analysis been completed, covering licensing, cloud services, hardware upgrades, model retraining, monitoring, and technical support across the full deployment lifecycle?	Medium	
	Has a postimplementation evaluation plan been defined (clinical impact, cost-effectiveness, unintended consequences)	High	

Each item was derived inductively from recurrent challenges and risks documented in the 100 included studies, ensuring that the checklist reflects real-world implementation experiences rather than theoretical considerations. To support practical use, two additional ratings were applied: maturity and priority.

By combining these two layers, IPC teams can gauge both how close their systems are to readiness and where to focus resources first. The maturity-priority framework thus transforms the checklist into a practical roadmap for planning, implementation, and risk mitigation.

The development of these scales followed a four-step, evidence-informed process:

1. Thematic synthesis: reviewers inductively coded implementation barriers and risks from the included studies and organized them into the six checklist domains.
2. Item drafting: recurring, actionable challenges within each domain were translated into checklist items.
3. Maturity anchors: the 4-level maturity scale was defined to reflect observable progression from non-existent capacity to embedded practice. This structure aligns with established digital health maturity concepts such as the Healthcare Information and Management Systems Society’s (HIMSS) Electronic Medical Record Adoption Model (EMRAM)^111^ and with implementation science frameworks on organizational readiness such as the Consolidated Framework for Implementation Research (CFIR).^112^
4. Priority criteria: item priority was determined using risk-management principles (safety and regulatory criticality, dependency and sequencing, feasibility and resource burden), aligned with two normative governance frameworks central to this review—the WHO guidance on ethics and governance of AI in health^6^ and the EU Artificial Intelligence Act,^7^ which classifies AI applications by risk. This ensures that high-priority items correspond to safeguards required for high-risk use cases.

The maturity scale was designed as a four-point ordinal measure:

- 0—Absent: no sign AI solution has been considered.
- 1—Emergent: early steps are present (e.g., draft policy, small pilot, budget line), but the system is not yet operational.
- 2—Functional: the system is operational but limited in scope or reliability.
- 3—Mature: the element is fully embedded, routinely monitored, and continuously improved.

This progression mirrors patterns observed in the literature, moving from complete absence of capacity (eg, no data stewardship), through pilots and partial functionality, to fully institutionalized, monitored systems. A neutral midpoint was deliberately excluded to encourage decisive appraisal of whether an element is operational or still requires attention.

The priority scale distinguishes between:

- High Priority items, which are essential for safe and effective AI use, such as complete data, model accuracy, cybersecurity, and legal compliance.
- Medium Priority items, which facilitate adoption and sustainability over time, including vendor support, return-on-investment data, or stress-testing of alert volumes.

## Discussion

This review maps how AI has been applied in IPC across 100 studies and distills common barriers and risks into a readiness checklist. Applications ranged from prediction to surveillance and compliance monitoring, but most systems remain at early stages. Success depends not only on algorithmic accuracy but on the conditions enabling reliable use: high-quality data, seamless integration, and organizational readiness. Three key messages emerge: data quality drives performance; integration is the main bottleneck; and barriers span technical, economic, and organizational domains.

### Data quality drives AI performance

According to our analysis, data completeness and quality influences AI systems performance and applicability. Across nearly every IPC application area, the most effective systems were built on complete, high-fidelity data. When microbiology codes, admission timestamps, and vital signs were reliably captured, predictive models for HAIs’ prevention such as surgical site infection (SSI) and *Clostridioides difficile* risk prediction routinely achieved high accuracy.^12,13,78^ Conversely, nearly half of all studies reported stalled or degraded performance due to missing fields, non-standard coding, or delayed data streams.^77,100^ From a practical standpoint, this suggests that data readiness must come before model adoption. Installing even the most sophisticated AI algorithm on top of fragmented or inconsistent inputs is unlikely to yield clinical value and may even introduce misleading noise.

### Digital integration is the real bottleneck

The study design distribution highlighted a pronounced translation gap: while experimental (30%) and retrospective investigations (49%) dominate, fewer than one-fifth of studies evaluate real-world implementation (17%), and rigorous external validation remains exceptional (4%). External validation or device-level verification is the step that exposes hidden overfitting, data-mapping errors, and workflow frictions before large-scale rollout. The scarcity of such studies therefore highlights a critical translational gap: most AI-for-IPC tools remain unproven outside their development sandbox. Moving the field beyond proof-of-concept will therefore require prospective, implementation-science designs that capture workflow integration, user acceptance, and downstream infection-control impact. The vast majority remained confined to isolated research platforms, often lacking connectivity with EHR interfaces, IPC dashboards, or real-time data streams from environmental or wearable sensors. However, by curating case definitions into formats interpretable by AI agents, even complex surveillance tasks such as the detection of HAIs, can be substantially automated. ^25,29,34,41,45,54,72,108^

Such frameworks could also be extended to support automated case-to-definition matching in outbreak investigations, reportable disease tracking, and clinical decision-making, particularly when embedded directly within electronic health records (EHR).^15,42,43,101^

Where digital integration did occur, for example, monitoring of PPE adherence or linking hand hygiene systems to IPC dashboards, teams were more likely to act on the outputs, leading to improved compliance and earlier intervention.^44,109^

Failures were often linked to technical incompatibilities (eg, software version mismatches after EHR upgrades) or infrastructure gaps, such as the absence of real-time IoT data pipelines but also due to high setup costs, requiring integration with existing systems for smooth workflow.^66,67,69^ This highlights a key insight: accuracy is not enough, usefulness depends on connectivity.

### Barriers compound across domains

Barriers rarely occur in isolation. Data gaps, fragile digital infrastructure, budget constraints, and users’ skepticism often interact, reinforcing one another. For instance, dependency on EHR structure and data quality and high computational cost contributed to model drift, which in turn produced irrelevant alerts that clinicians learned to ignore.^35,85^

Human factors, like alert fatigue or distrust, became more acute when paired with poor feedback loops or missing context.^102^ A simple AI model that functions transparently and within a familiar dashboard may be more sustainable than a black-box tool that overloads staff with unexplained alerts.

### Risk is not just technical, it’s operational

The risk landscape described across these studies suggests that operational vulnerabilities are at least as common, and potentially more disruptive, than algorithmic failures. In some studies, challenges were reported due to explainability of ML models and integration into daily routines due to complexity^59^ or misaligned response protocols.^31^ These risks affect patient care directly and erode staff trust.

Operational and technical risks frequently co-occurred, particularly in complex systems depending on high-quality data and computational power.^59^ Although relatively few papers discuss it (12%), data security such as patient privacy remain critical, especially as cloud-based IPC solutions scale across institutions combined with wireless solutions.

### Maturity levels vary across IPC application areas

Some IPC application areas appear closer to routine readiness. Predictive analytics (53%) and hand hygiene monitoring (13%) stand out: models often performed well, and compliance tools that provided immediate feedback were linked to tangible improvements.^12,13,17^ One pilot study demonstrated that a human-AI collaboration system accurately monitored PPE donning and doffing procedures in a simulated setting, suggesting that such systems could serve as a substitute or enhancement to in-person observers.^44,55^ These systems are beginning to move beyond pilot status in select settings. One study reported that despite some usability concerns, particularly related to AI system design and EHR integration, most users expressed overall satisfaction with AI-based hand hygiene monitoring.^68^ Importantly, the system was perceived to reduce HAIs and positively influence provider well-being, with younger and more experienced staff reporting greater satisfaction with AI use in direct patient care.^68^

By contrast, AMR prediction through WGS,^42,43^ and sensor-based environmental monitoring^27,69,106^ remain largely exploratory. Their value is conceptually clear, but technical complexity and cost remain barriers to widespread use.

For decision-makers, this suggests a tiered approach: focus first on AI tools with strong implementation evidence and realistic integration paths, while continuing to evaluate and pilot newer innovations with longer lead times.

AI tools already match or surpass conventional IPC approaches in prediction and compliance monitoring (eg, Hand Hygiene or PPE compliance) when high-quality data and robust integration are in place. However, data fragmentation, fragile interfaces, and unfunded maintenance obligations consistently limit scale-up. We also illustrate how these findings informed a readiness checklist that will offer IPC leaders a concise, evidence-based instrument to assess feasibility, allocate resources and mitigate risk before AI deployment.

#### Future directions

Few studies addressed explainability, equity, or sustainability. Post hoc explanation methods (eg, SHAP) were rare,^95^ and almost none reported energy or carbon footprints even if the Wash Ring project^109^ showed that efficiency gains are possible. Future research should combine performance metrics with explainability, sustainability reporting, and validation in diverse, low-resource settings.

## Limitations

Most included studies were single-center pilots from high-income countries (85%), limiting generalizability. Evidence on cost-effectiveness and patient outcomes was scarce. The proposed checklist is an initial synthesis that requires prospective validation and iterative refinement through consensus methods such as Delphi.

## Conclusion

Artificial intelligence offers real opportunities to strengthen infection prevention and control, from improving prediction and surveillance to supporting compliance monitoring. Yet most applications remain at an early stage, with progress slowed by gaps in data quality, weak system integration, and uneven readiness across healthcare settings. The evidence-informed checklist developed in this review is intended as a practical guide for IPC teams, helping them assess maturity, set priorities, and align implementation with international governance frameworks.^6,7^ Moving AI from promising pilots to routine practice will require reliable data, robust integration, and above all, clear accountability to ensure safe and effective use.

## Supporting information

10.1017/ash.2025.10191.sm001 Gastaldi et al. supplementary material 1 Gastaldi et al. supplementary material

10.1017/ash.2025.10191.sm002 Gastaldi et al. supplementary material 2 Gastaldi et al. supplementary material

## Data Availability

All data supporting the findings of this review are contained within the manuscript and its supplementary materials.

## Supplementary material

The supplementary material for this article can be found at https://doi.org/10.1017/ash.2025.10191.
