# Supplementary material for: Advancing infection prevention and control through artificial intelligence: a scoping review of applications, barriers, and a decision-support checklist
Source: Antimicrob Steward Healthc Epidemiol. 2025 Nov 25;5(1):e317. doi: 10.1017/ash.2025.10191 (PMC12722576; doi:10.1017/ash.2025.10191)
Supplement: Gastaldi et al. supplementary material 1 — Gastaldi et al. supplementary material [file S2732494X25101915sup001.docx]

Appendix A - Search Algorithm and PCC

| **Database** | **Query** | **Results** | **Search Date** |
| --- | --- | --- | --- |
| **PUBMED** | "infection prevention and control"[TIAB] OR "Cross Infection / prevention & control"[TIAB] OR "Infection Control / methods"[TIAB] OR "Surgical Wound Infection / prevention & control"[TIAB] OR "Hand Hygiene"[TIAB]) OR “Health acquired Infections” OR “Health care acquired infections”AND ("Artificial intelligence"[TIAB] OR "automated surveillance"[TIAB] OR "machine learning"[TIAB] OR "digital health"[TIAB] OR "predictive analytics"[TIAB] OR "healthcare technology"[TIAB] OR "AI-powered diagnostics"[TIAB] OR "wearable devices"[TIAB] OR "Internet of medical things (IoMT)"[TIAB] OR "tele-health"[TIAB] OR "cloud computing"[TIAB] OR "mobile health"[TIAB]) | 330 | 02/11/24 |
| **SCOPUS** | ({Infection prevention and control} OR {infection prevention} OR {Cross Infection} OR {Infection Control} OR {Surgical Wound Infection} OR {Hand Hygiene}) OR “Health acquired Infections” OR “Health care acquired infections” AND ({Artificial intelligence} OR {machine learning} OR {digital health} OR {automated surveillance} OR {predictive analytics} OR {AI-powered diagnostics} OR {digital innovations in IPC} OR {Automated monitoring} OR {emerging technologies} OR {blockchain} OR {Internet of medical things} OR {IoMT} OR {cloud computing} OR {mobile health} OR {e-health} OR {wearable devices} OR {tele-health} OR {Metaverse technologies} OR {virtual reality} OR {augmented reality} OR {mixed reality} OR {deep learning}) | 1234 | 01/11/24 |
| **WEB OF SCIENCE** | TS=({Infection prevention and control} OR {Infection prevention} OR {Cross Infection} OR {Infection Control} OR {Surgical Wound Infection} OR {Hand Hygiene}) OR “Health acquired Infections” OR “Health care acquired infections” AND TS=({Artificial intelligence} OR {Machine learning} OR {Digital health} OR {Automated surveillance} OR {Predictive analytics} OR {AI-powered diagnostics} OR {Digital innovations in IPC} OR {Automated monitoring} OR {Emerging technologies} OR {Blockchain} OR {Internet of medical things} OR {IoMT} OR {Cloud computing} OR {Mobile health} OR {E-health} OR {Wearable devices} OR {Tele-health} OR {Metaverse technologies} OR {Virtual reality} OR {Augmented reality} OR {Mixed reality} OR {Deep learning}) AND TS=({HEALTH CARE SETTING} OR {HEALTH FACILITY}) | 579 | 02/11/24 |

**PCC Framework**

- **Population**: Healthcare settings (e.g. hospitals, IPC units), and IPC professionals.
- **Concept**: Use of Artificial Intelligence (AI) technologies in support of Infection Prevention and Control (IPC), including predictive analytics, hand hygiene monitoring, HAI detection/surveillance, and other emerging applications.
- **Context**: Global healthcare systems, with attention to integration challenges, implementation risks, and decision-making tools (e.g., checklists) to support adoption.
